# Enhancing glioma treatment with 3D scaffolds laden with upconversion nanoparticles and temozolomide in orthotopic mouse model

**DOI:** 10.3389/fchem.2024.1445664

**Published:** 2024-10-21

**Authors:** Tatiana A. Mishchenko, Maria O. Klimenko, Evgenii L. Guryev, Alexander G. Savelyev, Dmitri V. Krysko, Sergey V. Gudkov, Evgeny V. Khaydukov, Andrei V. Zvyagin, Maria V. Vedunova

**Affiliations:** ^1^ Institute of Biology and Biomedicine, Lobachevsky State University of Nizhny Novgorod, Nizhny Novgorod, Russia; ^2^ Laboratory of Laser Biomedicine, NRC “Kurchatov Institute”, Moscow, Russia; ^3^ D. I. Mendeleev Russian University of Chemical Technology, Moscow, Russia; ^4^ Cell Death Investigation and Therapy Laboratory, Anatomy and Embryology Unit, Department of Human Structure and Repair, Faculty of Medicine and Health Sciences, Ghent University, Ghent, Belgium; ^5^ Prokhorov General Physics Institute of the Russian Academy of Sciences, Moscow, Russia; ^6^ Petrovsky National Research Center of Surgery, Moscow, Russia; ^7^ Department of Biomaterials and Bionanotechnology, Laboratory "Polymers for biology", Shemyakin-Ovchinnikov Institute of Bioorganic Chemistry RAS, Moscow, Russia; ^8^ Molecular Immunology Department, Shemyakin-Ovchinnikov Institute of Bioorganic Chemistry RAS, Moscow, Russia; ^9^ Institute of Molecular Theranostics, Sechenov First Moscow State Medical University, Moscow, Russia; ^10^ Scientific Center for Translational Medicine, Sirius University of Science and Technology, Sirius, Russia

**Keywords:** GL261 cells, hyaluronic acid hydrogels, upconversion nanoparticles, temozolomide, orthotopic glioma model

## Abstract

Targeted drug delivery for primary brain tumors, particularly gliomas, is currently a promising approach to reduce patient relapse rates. The use of substitutable scaffolds, which enable the sustained release of clinically relevant doses of anticancer medications, offers the potential to decrease the toxic burden on the patient’s organism while also enhancing their quality of life and overall survival. Upconversion nanoparticles (UCNPs) are being actively explored as promising agents for detection and monitoring of tumor growth, and as therapeutic agents that can provide isolated therapeutic effects and enhance standard chemotherapy. Our study is focused on the feasibility of constructing scaffolds using methacrylated hyaluronic acid with additional impregnation of UCNPs and the chemotherapeutic drug temozolomide (TMZ) for glioma treatment. The designed scaffolds have been demonstrated their efficacy as a drug and UCNPs delivery system for gliomas. Using the aggressive orthotopic glioma model *in vivo*, it was found that the scaffolds possess the capacity to ameliorate neurological disorders in mice. Moreover, upon intracranial co-implantation of the scaffolds and glioma cells, the constructs disintegrate into distinct segments, augmenting the release of UCNPs into the surrounding tissue and concurrently reducing postoperative damage to brain tissue. The use of TMZ in the scaffold composition contributed to restraining glioma development and the reduction of tumor invasiveness. Our findings unveil promising prospects for the application of photopolymerizable biocompatible scaffolds in the realm of neuro-oncology.

## 1 Introduction

Gliomas, which are primary solid tumors in the central nervous system, are highly malignant and prone to metastasis, accounting for about 70% of cases. They have an incidence rate of approximately 5 cases per 100,000 population, with a higher occurrence in individuals over 60 years of age. The majority of glial brain tumors (about 2/3) are found in the cerebral hemispheres (Larjavaara et al., 2007; Ostrom et al., 2022; Barnholtz-Sloan, Ostrom, and Cote, 2018). The infiltrative growth of these tumors and their initial location reduce the effectiveness of both primary treatments, which involve removing the primary tumor focus, and secondary treatments, which rely on radiation therapy. For highly malignant glial tumors, the most promising treatment option lies in subsequent chemotherapy (Stupp et al., 2005). However, even when the blood-brain barrier is compromised due to the development of the cancer, not all high-molecular-weight therapeutic agents can penetrate it. This limitation severely restricts treatment options in neuro-oncology.

In the past decade, attention has shifted towards methods for the delayed delivery of drugs, particularly the use of scaffolds that can be implanted during the resection of the primary tumor focus (Chew and Danti, 2017; Eslahi et al., 2021). The utilization of photopolymerizable composite materials offers a solution for the targeted delivery of low-drug doses. The development of scaffolds compatible with brain tissue, capable of controlled bioresorption with the release of antitumor agents, can enhance therapy effectiveness while significantly reducing the body’s toxic burden (Salagean et al., 2023). Previous efforts have explored various techniques for creating scaffolds compatible with brain tissue (Mishchenko et al., 2022; Mishchenko et al., 2018), with structured hyaluronic acid being identified as the most promising approach. Hyaluronic acid is a native structural component of the brain’s extracellular matrix which contributes to maintenance and homeostasis of the central nervous system (CNS) (Kochlamazashvili et al., 2010; Iaconisi et al., 2023; Krishnaswamy et al., 2019) and also positively affects tissue regeneration providing cell survival, appearance of newly generated neurons and synaptic plasticity in pathological states (Lainé, Brot, and Gaillard, 2022; Mishchenko et al., 2022; Shahi et al., 2020). In addition to its high biocompatibility with nerve cells which implies the minimization of toxic products formation during biodegradation, hyaluronic acid can be modified chemically by simple methods that enable cross-linking of polymer chains to highly tunable scaffolds (Djoudi et al., 2022; Jensen, Holloway, and Stabenfeldt, 2020; Spearman et al., 2020; Ahmadian et al., 2020). Photoinduced structuring of the material enables the creation of neurocompatible scaffolds with controlled properties and facilitates the use of complex, including high-molecular compounds as fillers (Mishchenko et al., 2022; Choi et al., 2019).

Besides scaffolds’ ability to maintain architectonics of nerve tissue (Mishchenko et al., 2022) at the site of tumor resection and their use as drug carriers, impregnation of theranostic agents broadens their functions in non-invasive monitoring of tumor growth and additional therapeutic effects that enhance standard chemotherapy. One of the highly promising tools for theranostics is upconversion nanoparticles (UCNPs). Various UCNPs are actively considered as activators of free radical processes in tumor cells (Wang et al., 2019). Moreover, they can serve as valuable agents for localizing tumor foci during computed tomography and magnetic resonance imaging scans (Borse et al., 2022; Ni et al., 2014), making them indispensable for image-guided therapy of inoperable tumors. Another significant approach to enhance the efficiency of UCNPs involves surface modification, often in conjunction with existing cytotoxic agents (Tian et al., 2015). Furthermore, UCNPs have demonstrated the capability to activate antitumor immunity (Zhu et al., 2023). These characteristics collectively highlight the potential of scaffolds as a means for the sustained delivery of UCNPs and other antitumor agents, offering a promising method for treating highly malignant gliomas.

Taking into account the abovementioned perspectives, the current work was devoted to the complex analysis of scaffolds impregnated with UCNPs and chemotherapeutic drug temozolomide (TMZ) on tumor growth progression upon simultaneous intracranial implantation of the scaffolds and viable glioma cells *in vivo*.

## 2 Materials and methods

### 2.1 Materials

The following materials were purchased from Sigma-Aldrich (USA): temozolomide (TMZ), sodium hyaluronate (Mn = 100 kDa), glycidyl methacrylate, tetraethylammonium bromide, poly (ethylene glycol) diacrylate (PEG-DA), poly (ethylene glycol) diglycidyl ether (PEG–DGE), poly(maleic anhydride-alt-1-octadecene) (PMAO), acetone, N,N-dimethylformamide, and potassium permanganate (KMnO_4_). Flavin mononucleotide was obtained from Pharmstandard, Russia and triethanolamine from Merck, USA. Phosphate buffered saline (PBS, pH 7.4) was prepared by dissolving a biotechnology grade PBS tablet (VWR Life Science, Canada) in 100 mL of deionized water. Penicillin-streptomycin (5000 U/mL and 5,000 μg/mL, respectively) was purchased from PanEco (Russia). Amphotericin B (5,000 μg/mL) was purchased from JSC “Sintez” (Russia). Murine glioma GL261 cell were kindly provided by Prof. Dr. P. Agostinis (Laboratory of Cell Death Research and Therapy, Department of Cellular and Molecular Medicine, KU Leuven, Belgium). For cell culturing, Dulbecco’s Modified Eagle’s Medium (DMEM), sodium pyruvate, penicillin-streptomycin, L-glutamine and Ca^2+^- and Mg^2+^-free phosphate-buffered saline (PBS) were purchased in Thermo Fisher Scientific (USA); fetal bovine serum (FBS) was obtained from Biosera, France. Trypsin-EDTA (0.25%) and versine solution were supplied by PanEco, Russia. Seventy adult male C57BL/6 mice aged 5–7 weeks were housed in a certified SPF vivarium of Lobachevsky University. For general anesthesia, isoflurane was purchased in Laboratorios Karizoo, Spain; a dental composite was supplied by DentLight-flow, Russia. Metacam for post-operative analgesia was purchased in Boehringer Ingelheim, Germany. For morphological assessment, cryogel was purchased in Leica, Germany and hematoxylin-eosin reagents were obtained from PanReac AppliChem, Germany; the mounting medium was supplied by Thermo Fisher Scientific, USA.

### 2.2 Synthesis of UCNPs

UCNPs of the core/shell composition NaY_0,794_Yb_0,2_Tm_0,006_F_4_/NaYF_4_ were synthesized via solvatothermal decomposition with additional thermal treatment (∼310 ^0^С). This process facilitated the attainment of a more stable β-hexagonal phase. Detailed synthesis procedures for UCNPs can be found in previous publications (Mironova et al., 2017; Mishchenko et al., 2020). The trivalent lanthanide ions, ytterbium Yb^3+^ and thulium Tm^3+^, were used for doping the NaYF_4_ matrix. This resulted in the generation of UCNPs exhibiting photoluminescence emission maxima in both the blue region (474 nm) and the infrared region (801 nm) when excited with 980 nm light. Upconversion is a nonlinear optical process where a nanoparticle sequentially absorbs two or more low-energy photons and emits a high-energy photon of a shorter wavelength (Hodak et al., 2016; Liu et al., 2017; Wang et al., 2008).The conversion ratio of the obtained UCNPs upon irradiation with infrared (IR) light of 1 W/cm^2^ was 2%, indicating high brightness of photoluminescence. The presence of the excitation and emission maxima of UCNPs photoluminescence in the IR region is a fundamentally necessary characteristic for visualizing material in a biological tissue transparency window that provides monitoring of its resorption over time (Mishchenko, Balalaeva, et al., 2022; Kostyuk et al., 2019). Previous exhaustive study of the general and specific toxicity of UCNPs including assessment of their allergenic, immunotoxic, and reprotoxic properties, strongly supports the notion that UCNPs are functional, noncytotoxic, biocompatible, and safe for imaging applications in cells, small animals, and prospective clinical applications (Guryev et al., 2019). In the model system *in vitro*, we have shown the absence of pronounced cytotoxic effects of UCNPs against glioma GL261 and mild cytotoxicity for normal brain cells in the remote period after UCNPs application (Mishchenko et al., 2020).

The UCNPs were coated with an additional shell of an amphiphilic alternating copolymer PMAO to ensure effective surface hydrophilization. PEG–DGE was used to further stabilize the shell structure of UCNPs. The hydrodynamic diameter of the UCNPs coated with PMAO was 119 ± 9 nm with a polydispersity index (PDI) of 0,66. After PEG-DGE addition, the average hydrodynamic diameter of UCNPs decreased to 75 ± 15 nm at PDI of 0.136, indicating the shell compaction and UCNPs colloidal stabilization.

### 2.3 Scaffolding with UCNPs and temozolomide impregnation

The design of scaffolds was based on methacrylated hyaluronic acid processed into a photocurable composition. Our previous studies *in vitro* and *in vivo* have shown that scaffolds based on methacrylated hyaluronic acid possess a favorable biocompatibility with brain cells and provide an opportunity to facilitate the loading of soluble therapeutic or biologically active agents (Mishchenko, Klimenko, et al., 2022).

Hyaluronic acid (1 g) was dissolved in 200 mL of deionized water with subsequent addition of 120 mL of dimethylformamide. Tetraethylammonium bromide (1 g) was used in the reaction mixture as an interfacial transfer catalyst to improve glycidyl methacrylate GMA solubility. Penicillin-streptomycin (5000 U/mL and 5,000 μg/mL) and amphotericin B (5,000 μg/mL) were added to minimize the risk of contamination. After complete dissolution of these components, 14 mL of HAGM was added while maintaining continuous stirring at 25°C. Hyaluronic acid modified with GMA (HAGM) was isolated through precipitation in a seven-fold excess of acetone followed by purification by dialysis against distilled water and lyophilization in a FreeZone Freeze Dryer (Labconco, United States). The degree of substitution (DS) of disaccharide units of hyaluronic acid with GMA was measured according to the previously described protocol of colorimetric reaction of double bonds with potassium permanganate (Sochilina et al., 2019). The photocurable composition was produced by dissolving GMA (DS 36.6% and DS 47.3% in a 1:1 ratio) in an isotonic 0.9% NaCl solution. Flavin mononucleotide (0.001 wt%) with triethanolamine (0.5 wt%) was added as a photoinitiating complex. A non-toxic linear polymer PEG-DA (Mn = 575, 2.5 wt%) was added for optimization of the mechanical properties of hydrogels. Temozolomide or UCNPs were mixed into the solution at the resulting concentration of 0.67% and 85–88 μg/mL, respectively. The mixture was then dissolved in an ultrasonic bath until a uniform consistency. Gelation of the photocurable composition was accomplished through light excitation with a wavelength of 450 nm and an intensity of 480 mW/cm^2^ using a Vega-Solaris diode (Polironik LLC, Russia).

### 2.4 Glioma cell culture

Murine glioma GL261 cells were cultured in DMEM medium containing 4.5 g/L glucose supplemented with 10% FBS, 2 mM L-glutamine, 100 µM sodium pyruvate, 100 U/mL penicillin and 100 μg/L streptomycin. The viability of cells was maintained at 37^0^C in a humidified atmosphere containing 5% CO_2_ in a Binder C150 incubator (BINDER GmbH, Germany). The cells of subconfluent state were reseeded at an approximate cell density of 1.0 × 10^5^ cells/mL using a trypsin–versene solution (1:3). The glioma cells for orthotopic inoculation were taken after the third passage.

### 2.5 *In vivo* orthotopic transplantation of glioma GL261 cells and scaffolds implantation

Seventy male C57BL/6 mice aged 5–7 weeks were employed in this study. The mice were housed in a certified SPF vivarium of Lobachevsky University. The mice received general anesthesia with isoflurane (5% in a gas mixture for induction and 1.5%–2% for maintenance). In the absence of proprioceptive reflexes, the mice’s head was cleared of fur and fixed in a stereotactic frame (World Precision Instruments, USA). The soft tissue was dissected, and a trepanation window in the right hemisphere was formed with a fine drill following the stereotactic coordinates (2 mm lateral and 2 mm posterior to the bregma). Glioma GL2621 cells (3 × 10^4^ cells in 2 µL PBS) and scaffold (2 mm × 2 mm x 2 mm) were placed into the brain tissue in an approximate depth of 2 ± 0.5 mm. To allow the real-time monitoring of the propagation of fluorescent signals from UCNPs, the trepanation window was sealed with a sterile cover glass, affixed to the skull using a dental composite that was solidified using a LUX V lamp (Woodpecker/DTE, China). The wound was then closed with adjacent tissues tightly sutured with surgical thread (0.2 mm) and were treated with antiseptic to avoid infection. Metacam (0.002 mg/mL) was given for post-operative analgesia. Following recovery from anesthesia, the animals were returned to their cages and granted postoperative care with *ad libitum* access to food and water.

**FIGURE 1:**
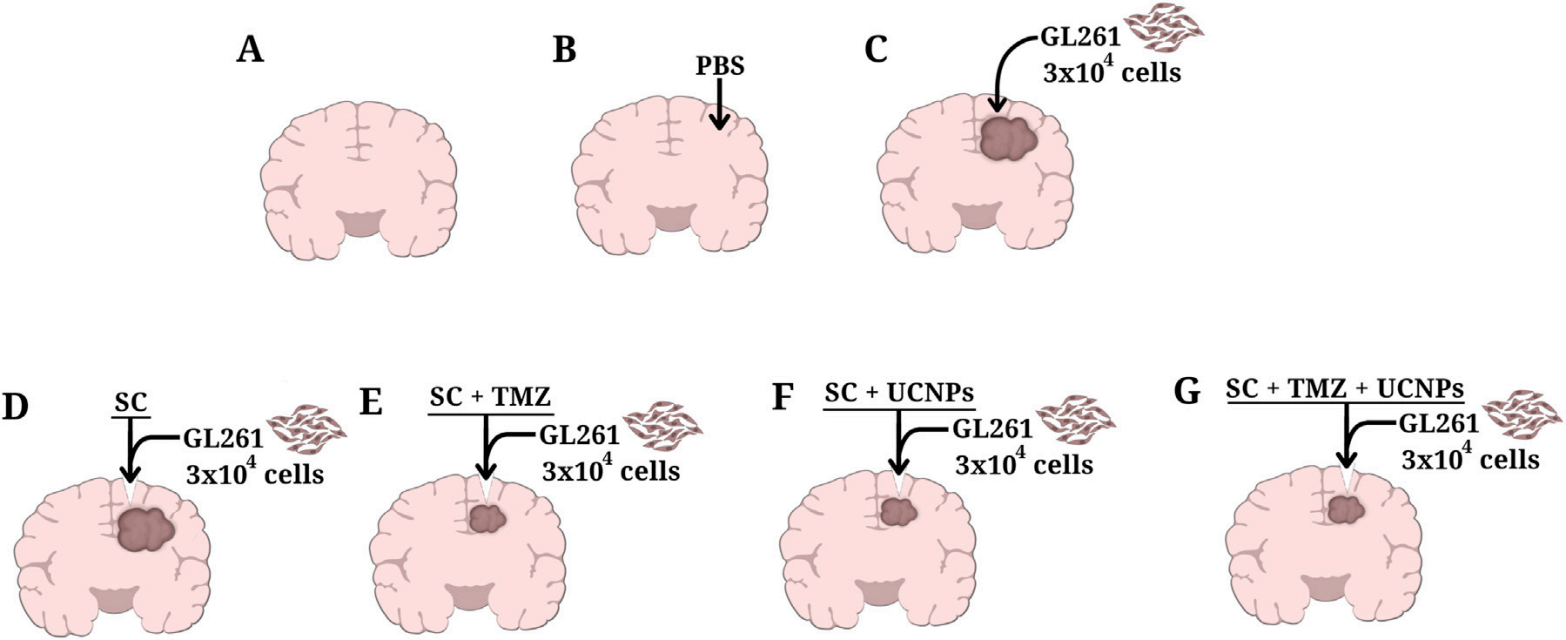
Schematic representation of control and experimental groups of C57BL/6 orthotopic glioma mice model used in the study. **(A)** Intact, **(B)** Intact+PBS, **(C)** Control, **(D)** SC, **(E)** SC+TMZ, **(F)** SC + UCNPs, **(G)** SC + TMZ + UCNPs.

The mice were divided into the following groups (Figure 1).

A) Intact–mice that did not undergo surgical procedures (n = 10);
B) Intact + PBS–mice with intracranial injection of phosphate-buffered saline (PBS, 2 µL) (n = 10);
C) Control–mice with intracranial injection of glioma GL261 cells (3 × 10^4^ cells in 2 µL PBS) (n = 10);
D) SC–mice with intracranial injection of glioma GL261 cells followed by implantation of scaffold devoid of UCNPs and TMZ (n = 10);
E) SC + TMZ–mice with intracranial injection of glioma GL261 cells followed by implantation of scaffold impregnated with TMZ (resulting concentration of the active substance 0.67%) (n = 10);
F) SC + UCNPs–mice with intracranial injection of glioma GL261 cells followed by implantation of scaffold impregnated with UCNPs (resulting concentration of 85–88 μg/mL) (n = 10);
G) SC + TMZ + UCNPs–mice with intracranial injection of glioma GL261 cells followed by implantation of scaffold impregnated with UCNPs and TMZ (n = 10).

Within 2 weeks after glioma cells inoculation and scaffold implantation, the mice were evaluated for neurological status, general locomotor and orienting-exploratory activity in the “Open Field” setup, as well as cognitive functions and learning ability in the passive avoidance test.

### 2.6 Neurological status assessment

The functional state of the CNS was assessed using a neurological deficit severity scale adapted for mice (Mishchenko, Klimenko, et al., 2022; Mishchenko, Balalaeva, et al., 2022; Mishchenko et al., 2023). This scale consisted of ten tests that evaluated parameters such as motor activity, coordination of movements, muscle tone, reflexes, ptosis, and exophthalmos. Each test was scored with two points for no reaction, one for some disturbances, and 0 for normal reaction. The points obtained were summarized and interpreted according to the following grade: severe CNS damage (10–20 points), moderate CNS damage (six to nine points), and light CNS damage (one to five points).

### 2.7 Open field test

The general locomotor and orienting-exploratory activity of the mice was tested in the Open Field Box (LE800S; Panlab Harvard Apparatus, Spain). Each animal was placed in the arena center with subsequent recording its behavioral reactions using a Sony SSC-G118 camera (Tokyo, Japan) for 5 min. The following parameters were then analyzed: general motor activity (number of the squares crossed in the arena), vertical motor activity (number of upright postures), and emotional state (time spent in the center of the arena, number of grooming acts, and acts of defecation and urination).

### 2.8 Passive avoidance test

To evaluate the learning ability of mice we applied a passive avoidance test using a Shuttle Box LE918 setup (Panlab Harvard Apparatus, Spain). The training session lasted 180 s. The animal was placed in the lighted section of the setup, and the time before animals entering to the dark section was measured. An electric stimulus (0.08 mA) was applied 5 s after the mouse entered the dark section. The retesting session lasting 300 s was performed 24 h after the training session (Mishchenko et al., 2022).

### 2.9 Histological analysis

For morphological assessment, the brains were surgically isolated and fixed in 10% formalin solution at room temperature for 24 h. The brain samples were then incubated at room temperature in 15% sucrose solution (24 h) followed by incubation in 30% sucrose solution (24 h). Next, the samples were gradually filled with cryogel, and cut into 15-μm coronal sections using a Leica CM1520 freezing sliding cryostat (Leica, Germany). The sections obtained were then subjected to hematoxylin-eosin staining. After dehydration in alcohol solutions of increasing concentrations with subsequent purification in xylene, the sections were embedded in mounting medium and examined using a Zeiss Primo Star light microscope (Zeiss, Germany) with an integrated Axio CamMRc camera (Zeiss, Germany).

### 2.10 Statistical data analysis

The data were statistically processed using GraphPad Prism (v.9.3.1.471) (San Diego, CA, USA). The results are presented as the mean ± standard error of the mean (SEM). The Shapiro–Wilk test was used for normal distribution analysis. We performed the Mann-Whitney test or the Wilcoxon t-test. The one-way ANOVA test was employed for statistical data analysis of the conditioned passive avoidance reflex reproduction. The difference was considered to be statistically significant if *p* < 0.05.

## 3 Results

### 3.1 Investigation of *in vivo* bioimaging of scaffolds using luminescent properties of UCNPs

**FIGURE 2:**
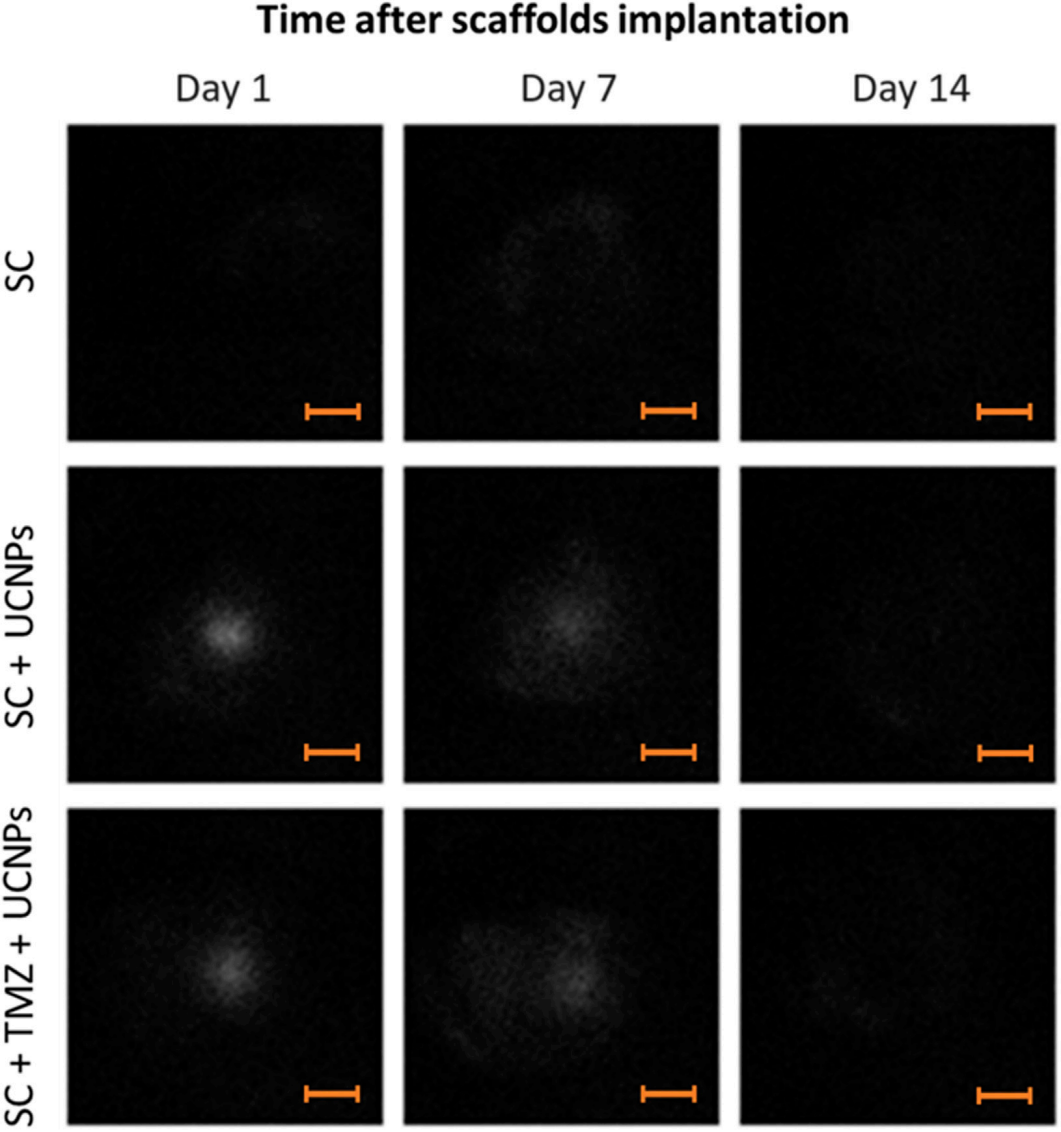
Photoluminescent imaging assessment of mouse brains after implantation of scaffolds loaded with UCNPs. Scale bars – 3 mm.

In the initial part of the study, we assessed the capability to visualize scaffolds impregnated with UCNPs after implantation in the mouse brain. To perform a photoluminescent imaging *in vivo*, a whole-body imaging system built in-house (Deep Vision System, DVS-02, arranged for anti-stokes luminescence detection) was used. Photoluminescent images of the animals were obtained in the range of 485–831 nm under excitation at 980 nm with external semiconductor laser module ATC-C4000-200AMF-980-5-F200 (Semiconductor Devices, Russia). The excitation light power was 0.77 W, the exposure time was 60 s. A detailed description of the imaging system is given in Supplementary Figure S1. Image analysis was performed using ImageJ 1.47v software (National Institute of Health, USA). Photoluminescent images were acquired on days 1, 7, and 14 after implantation. We demonstrated effective visualization of scaffolds in the SC + UCNPs and SC + TMZ + UCNPs groups with sufficiently high contrast (up to 4) during the observation period (Figure 2). Quantitative analysis of the photoluminescent signal in the area of scaffold implantation showed no decrease in intensity during 7 days after surgery. The photoluminescent signal was uniformly distributed across the entire region of the surgical window. However, on day 14 after scaffold implantation, no distinct photoluminescent signal was detectable in any of the mouse groups.

**FIGURE 3:**
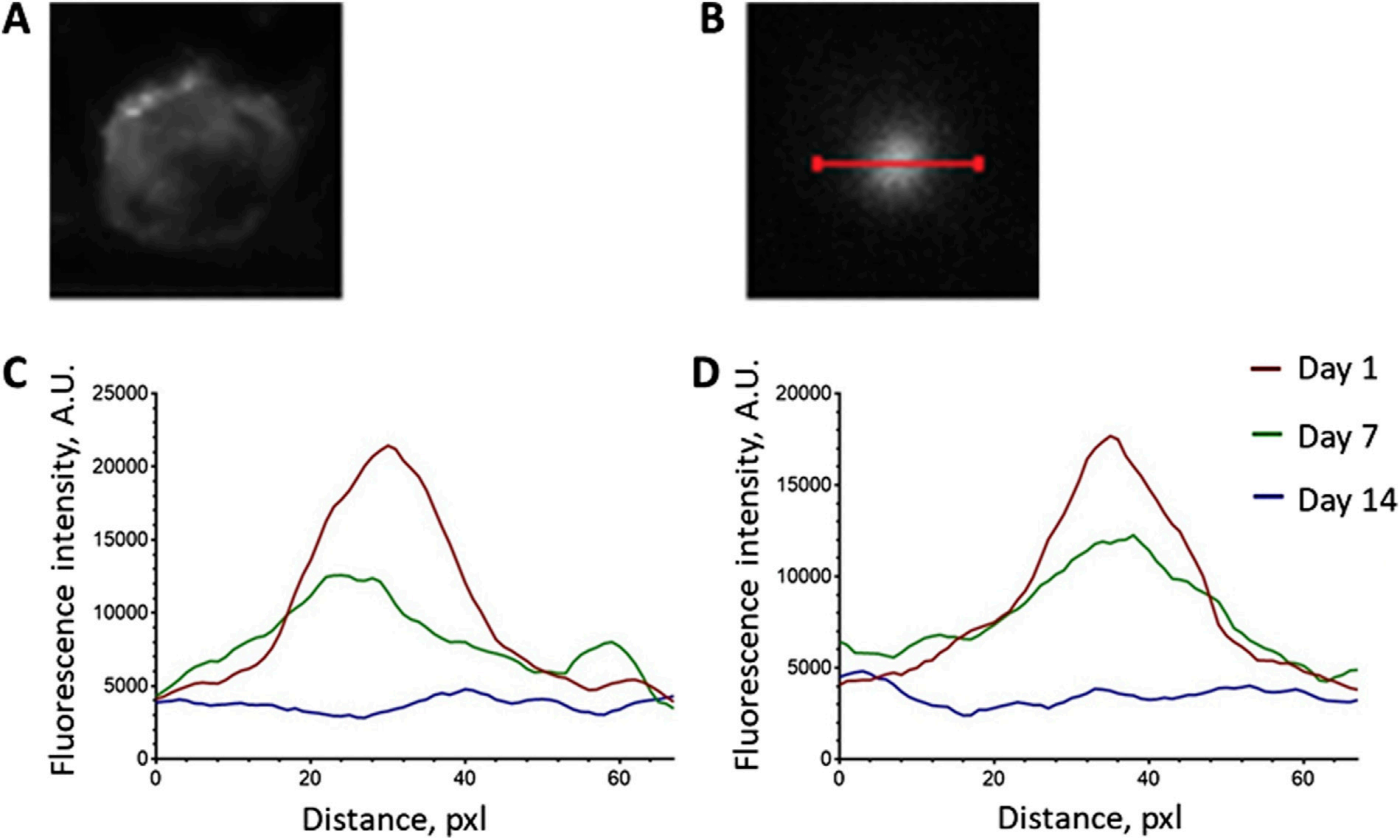
Analysis of the photoluminescence intensity profiles of the UCNPs signal along the line crossing the operating window. **(А)** operating window image; **(B)** photoluminescent image of the operating window and the line location; **(C)** representative intensity profile of SC + UCNPs photoluminescent signal along the selected line; **(D)** representative intensity profile of SC + TMZ + UCNPs photoluminescent signal along the selected line.

To quantify the scaffold resorption rate, we analyzed the photoluminescence intensity profiles of the UCNPs signal along a line crossing the operating window (Figures 3A–D). As can be seen from the graphs (Figures 3C, D), there is a decrease in the maximum values of photoluminescence intensity and more uniform signal distribution along the selected segment 7 days after scaffolds implantation. Taking into account that large UCNPs are not able to freely exit the scaffolds in contrast to low molecular weight substances (including TMZ), such signal redistribution suggests a gradual resorption of the constructs after implantation. The absence of a clear photoluminescence signal on the 14th day indicates the partial destruction of the scaffold structure and the release of UCNPs into the adjacent tissues. These findings underscore the practical feasibility of monitoring the preservation of implanted scaffold structures through the use of the photoluminescent properties exhibited by UCNPs.

### 3.2 The effects of scaffolds impregnated with UCNPs and TMZ on glioma growth progression

#### 3.2.1 Assessment of sensorimotor and behavioral reactions of mice after glioma cells inoculation and scaffolds’ implantation

**FIGURE 4:**
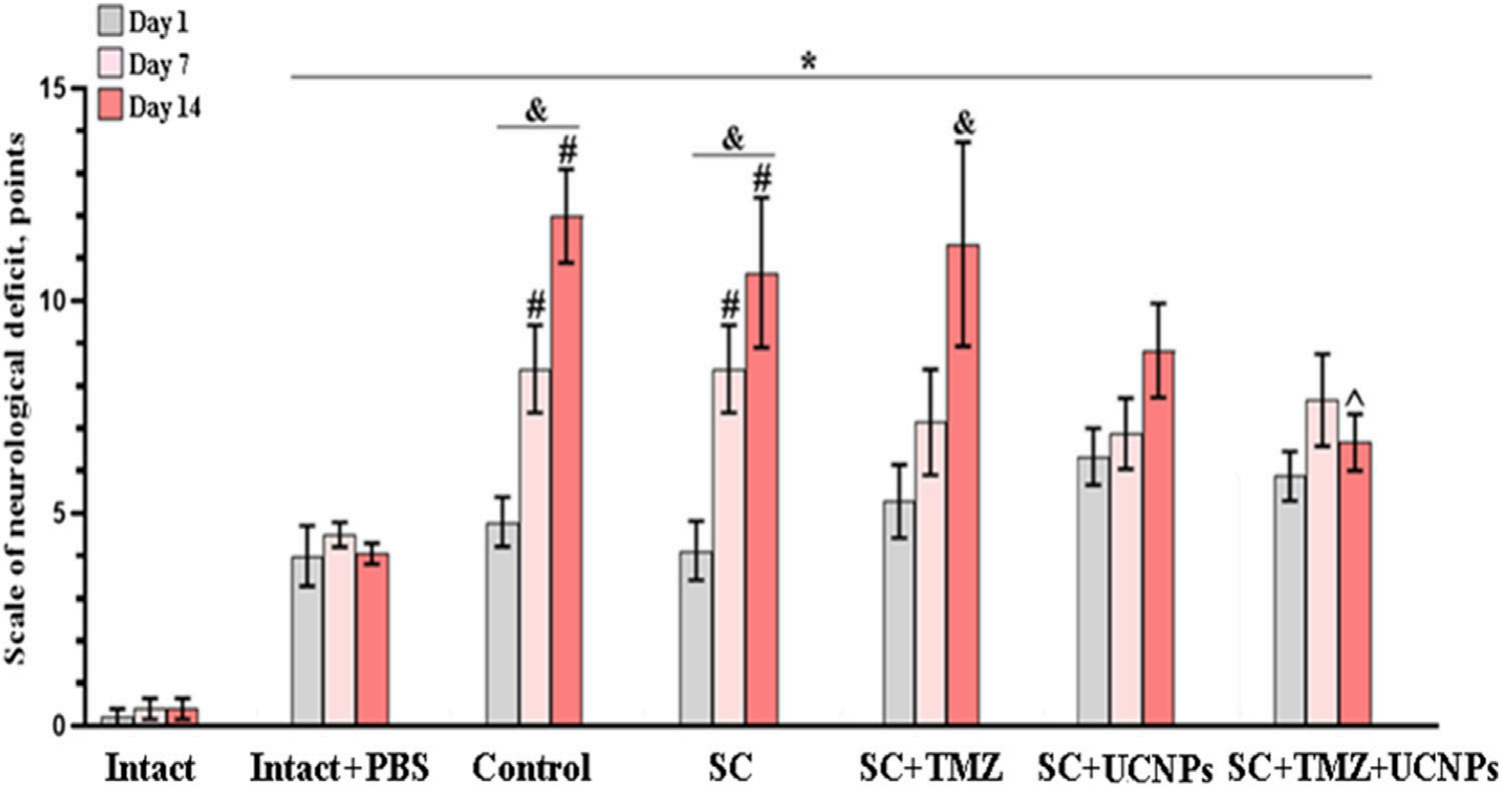
Neurological status assessment in mice after inoculation of glioma GL261 cells and implantation of scaffolds. The data demonstrate a decrease in the intensity of neurological tension development in mice implanted with scaffolds impregnated with UCNPs or combination of UCNPs and TMZ during 2 weeks after surgery. * – vs*.* “Intact”, # – vs*.* “Intact + PBS”, ^ – vs*.* “Control”, р < 0.05, the Mann-Whitney test, and – vs*.* first day after inoculation, р < 0.05, the Wilcoxon t-test, n = 6–10.

The neurological status assessment revealed a gradual development of neurological deficits in mice during 2 weeks after inoculation of glioma GL261 cells (Figure 4). In the control group, the mean scores of neurological deficits on day 7 after inoculation was 8.4 ± 1, indicating moderate CNS damage. Pronounced ptosis and deficits in the proprioception of the body was manifested by changes in postural stability and asymmetry of movements. By the end of the following week (day 14), the scores reached levels indicative of severe CNS damage, with a mean score of 12 ± 1 points.

Similar dynamics was observed in mice from the SC and SC + TMZ groups. The animals exhibited weak motor activity and decreased hind limbs responses to mechanical stimuli by day 10 after glioma cells inoculation. The values of the neurological deficits were reached severe lesions of the CNS by day 14 after glioma cells inoculation and amounted to 10.6 ± 1.7 (SC) and 11.3 ± 2.4 (SC + TMZ) points, respectively.

In contrast, mice implanted with scaffolds impregnated with UCNPs showed less intense development of neurological deficits. On day 14 post-implantation, the average neurological deficit score was 8.8 ± 1.1, corresponding to moderate CNS damage. These mice exhibited pronounced ptosis, weak motor activity, mostly mediated by stretching of forelimbs and their weakness. The use of scaffolds impregnated with UCNPs and TMZ contributed to a decrease in the intensity of neurological tension development in mice. On day 14 after glioma cells inoculation, the mean scores of the severity of the neurological deficits in the SC + TMZ + UCNPs group corresponded to moderate CNS damage (6.6 ± 0.6) and were significantly lower compared to those in the control group.

The analysis of behavioral reactions in the Open field test revealed significant changes in the locomotor activity and orienting-exploratory activity of mice on day 14 after glioma cells inoculation and implantation of scaffolds (Table 1).

**TABLE 1:** Parameters of behavioral reactions of mice in the Open Field test on day 14 after glioma GL261 cells inoculation and scaffold implantation.

A: Parameters of locomotor activity				
Group	Number of squares passed in the arena		Time in the arena center [s]	Number of upright postures
	Periphery	Center		
Intact	96.2 ± 5.5	21 ± 3.7	29.8 ± 6.3	11.2 ± 3
Intact + PBS	109.6 ± 8.9	16 ± 2.6	26.6 ± 5.9	11 ± 3.5
Control	68.7 ± 28.3	29 ± 3.8	127.3 ± 57.9*	2 ± 0.9*
SC	72.3 ± 25.2	17.3 ± 4	80 ± 35.2	2.3 ± 1.8*
SC + TMZ	51 ± 25.7	8 ± 2.6*	142.3 ± 79.3	2.3 ± 1.4*
SC + UCNPs	61.5 ± 20.6	22 ± 5.3	113.7 ± 46.3*	1.25 ± 0.75*
SC + TMZ + UCNPs	55.3 ± 28	6.6 ± 3.8*	141.6 ± 80.1	0.7 ± 0.2*

B: Emotional status characteristics				
Group	Time of grooming, s	Number of peeks in holes	Acts of urination	Acts of defecation
Intact	1 ± 0.6	41 ± 3.6	0 ± 0	0 ± 0
Intact + PBS	2.3 ± 2	24.6 ± 3.5	0 ± 0	0 ± 0
Control	0 ± 0	10.7 ± 4.2*	0 ± 0	0 ± 0
SC	4.6 ± 3.7	10.3 ± 2.6*	0 ± 0	0 ± 0
SC + TMZ	0.7 ± 0.6	12 ± 5.3*	0 ± 0	0 ± 0
SC + UCNPs	8.25 ± 5.45	15.25 ± 4.8*	0.25 ± 0.1	0 ± 0
SC + TMZ + UCNPs	0 ± 0	10.6 ± 4*	0 ± 0	0 ± 0

* - *v*s. “Intact”, р < 0.05, the Mann-Whitney test.

The mice in the control group spent the increased time in the center of the arena and showed a decreased orienting-exploratory activity, characterized by a significant decrease in the number of upright postures and peeking into the arena holes; the absence of grooming acts also indicated emotional tension in the animals. These pronounced changes in the behavioral reactions of mice correlated with the development of a significant neurological deficit.

Low orienting-exploratory activity and emotional tension were observed in mice of all experimental groups. The preferred behavioral strategy of mice implanted with scaffolds of various compositions was to stay in the center of the arena when introduced to a novel environment. The mice of the SC + TMZ and SC + TMZ + UCNPs groups showed increased stress load–the animals in the center of the arena exhibited decreased motor activity relative to the intact group.

#### 3.2.2 Analysis of cognitive abilities of mice after glioma cells inoculation and scaffolds implantation

**FIGURE 5:**
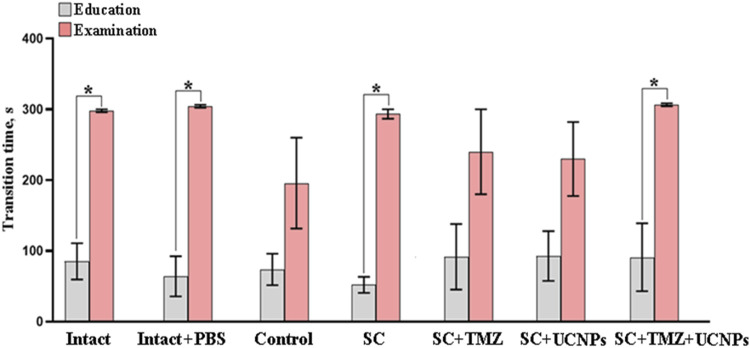
Evaluation of the conditioned passive avoidance reflex reproduction in mice on day 14 after inoculation of glioma GL261 cells and implantation of scaffolds with different compositions. The data show that implantation of control scaffolds as well as scaffolds impregnated with UCNPs and TMZ contributed to the preservation of the animals’ learning abilities and had no significant effect on cognitive functions. * –*vs* training session, р < 0.05, the one-way ANOVA test, n = 6–10.

The effect of inoculation of glioma cells and scaffolds implantation on cognitive functions and learning ability of mice was analyzed using the passive avoidance test (Figure 5). It was shown that active tumor growth led to pronounced impairments of cognitive abilities in mice. During the training session, the mice of the intact group entered the dark chamber section and tended to leave the brightly lit space, following the mink reflex. During the retest session, the latent time of movement to the dark section was significantly increased suggesting the formation of a memory trace in the mice. In the control group, the latent time of movement to the dark section in the training session did not differ from the latent time in the retest. A similar pattern of changes was observed in mice implanted with scaffolds containing UCNPs or TMZ. Implantation of control scaffolds (SC) as well as scaffolds impregnated with UCNPs and TMZ (SC + TMZ + UCNPs) contributed to the preservation of the animals’ learning abilities and had no significant effect on cognitive functions. The duration of the latent period in the SC and SC + TMZ + UCNPs during the retest session was significantly increased by 5.7 and 3.3 times, respectively, compared to the training session; these values did not differ from those of the intact group. Delayed motor reaction and selection of a safe section of the chamber characterize the normal process of memory formation and learning in the mice.

#### 3.2.3 Peculiarities of the brain tissue morphology after glioma cells inoculation and scaffolds implantation

**FIGURE 6:**
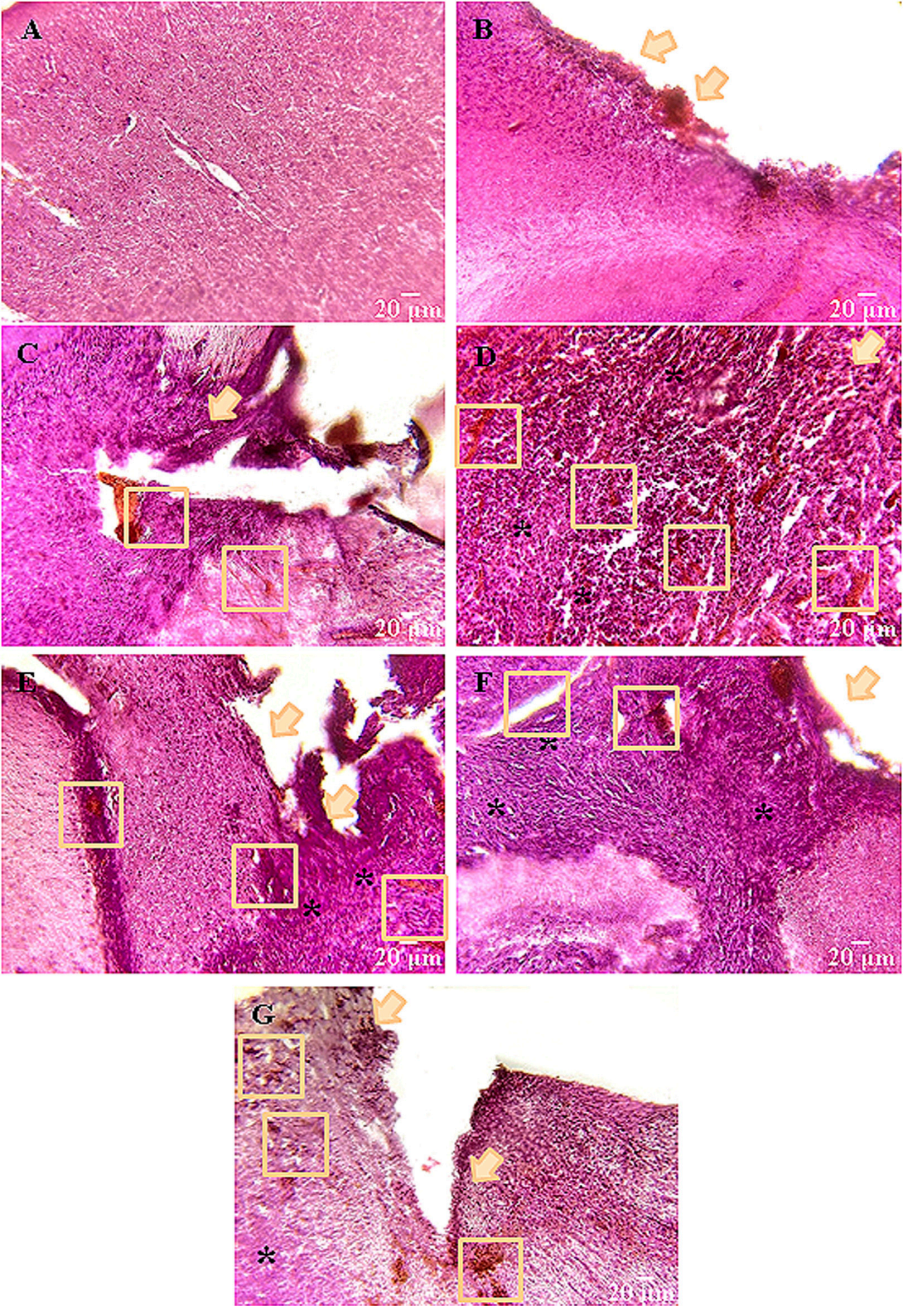
Representative histological samples of brain cortex of mice on day 14 after glioma GL261 cells inoculation and scaffolds implantation. **(А)** Intact, **(B)** Intact + PBS, **(C)** Control, **(D)** SC, **(E)** SC + TMZ, **(F)** SC + UCNPs, **(G)** SC + TMZ + UCNPs. All images were selected from approximately the same areas of damage. Orange arrows indicate the main focus of inflammation–the main site of tissue restructuring. The ruptures and vascular weaves are enclosed by orange squares; * denote the areas of tumor cell invasion. Hematoxylin-eosin staining (magnification ×20; scale bars – 20 µm).

Histological assessment revealed a typical brain cortex morphology of the intact mice (Figure 6). Neurons of regular round or oval shape with numerous outgrowths were visualized; cell nuclei were elongated-oval in shape with clear borders, occupying about half of the total cell volume; cell cytoplasm was homogeneous, without atypical inclusions, the structure of the vascular system was without pathologies.

In the Intact + PBS group, a shift of the nuclear-cytoplasmic ratio towards the increase of the nucleus volume, and tissue ruptures of small volumes (3–4 cases in 10 fields of view) were visualized near the site of PBS injection. There were single foci of necrosis, typically with a point location (Figure 6).

Orthotopic inoculation of glioma GL261 cells caused significant morphological changes in the brain cortex of mice. The control group was characterized by a complete loss of brain tissue structure. The right hemisphere was significantly shifted towards the left hemisphere, and the tumor site exhibited an irregular shape with numerous ruptures and large vascular tangles. The tumor volume was 2.9–3.1 mm^3^ on average. Tumor cells had a typical large nucleus often with an elongated shape. In deep layers of brain tissue, as well as in the area of tumor cell injection, pronounced foci of necrotic masses and apoptotic processes are visualized (Figure 6).

Similar dynamics of changes was observed in the groups where scaffolds were implanted; however, the tumor focus was shifted to the lower layers of the brain. Notably, a significant portion (70%) of the tested samples lost their structure. The largest tumors were detected in the SC (2.4–2.5 mm^3^) and SC + UCNPs (1.8–2.1 mm^3^) groups. Hemorrhagic areas were observed on the surface of the cerebral cortex. Tissue lysis predominated at the borders with the tumor bed, contributing to the formation of structureless matter. This process stimulated the formation of foci of tissue overgrowth and its pre-necrotic changes. Individual secondary tumor foci were visualized in 10 fields of view. In the SC + UCNPs group, a dense glial scar has formed around the deepest outgrowths of tumor cells.

Weak positive dynamics were observed in the groups with implantation of scaffolds loaded with TMZ. The invasive tumor growth in the SC + TMZ and SC + TMZ + UCNPs groups was less intense compared to the other experimental groups. Histological samples indicated a clear transition between tumor cells and nervous tissue parenchyma mediated by a small glial scar. The tumor volume was reduced and averaged to 1.6–2 mm^3^ SC + TMZ and 1.4–1.6 mm^3^ SC + TMZ + UCNPs, respectively. On the other hand, areas of angiogenesis and hemorrhage were found. Near the tumor bed, two to three weakly expressed necrotic sites were observed. Below the site of scaffold implantation, there were individual small outgrowths of tumor cells and the average total area of inflammation is reduced.

## 4 Discussion

In our study, we employed the GL261 glioma cell line, known for its highly aggressiveness (Szatmári et al., 2006), to simulate active tumor growth and metastasis. The primary antitumor agents under consideration included UCNPs and their combination with temozolomide. TMZ stands as a first-line chemotherapeutic drug for treating highly malignant gliomas across all age groups. While it exhibits a marked cytostatic effect similar to many alkylating agents and the ability to cross the blood-brain barrier due to its small molecular size, TMZ is accompanied by significant toxicity and often presents challenges for patients (Scaringi et al., 2013; Arora and Somasundaram, 2019). By using a scaffold based on methacrylated hyaluronic acid for targeted delivery of low doses of the drug, it becomes possible to mitigate the adverse systemic impact of TMZ and amplify its therapeutic effects. Our study revealed that even when used at low concentrations, TMZ displayed a noteworthy cytotoxic effect against glioma cells. Moreover, controlled photoinduced cross-linking of hyaluronic acid may, in the future, enable the creation of scaffolds with bioresorption rates approximating those of full courses of postoperative chemotherapy. These developed scaffolds not only offer unique mechanical properties, attributed to the use of non-toxic photoinitiators, specifically riboflavin, but have also demonstrated unique biocompatibility, as previously demonstrated (Mishchenko et al., 2018; Mishchenko, Klimenko, et al., 2022). Furthermore, the photoinduced gelation technology permits the creation of personalized scaffolds at the moment of primary tumor focus removal.

An intriguing observation is that the use of scaffolds, both alone with UCNPs and in combination with TMZ, resulted in a reduction of neurological deficits in the experimental animals. When characterizing the tumor process, it is crucial not only to consider the tumor’s size but also to examine the characteristics of cancer-transformed cells’ distant influence on brain tissue (Zhang and Yu, 2019; Ghaemmaghami et al., 2020). Even in the aggressive GL261 model, we observed a decrease in infiltrative tumor growth with the administration of UCNPs and TMZ. The formation of a clear barrier between tumor and normal nerve tissue by a small glial scar suggests deterrence of infiltrative tumor growth and neuroprotection of brain cells supporting their functional activity. Such adaptive mechanisms, discernible in histological sections, had a pronounced physiological impact on the evaluation of neurological deficits and memory functions. This impact might be associated with altered glioma cell effects on the activity of brain neurons (Venkatesh et al., 2019; Krishna et al., 2023). The reduction in infiltrative tumor growth and the mitigation of both distant and direct effects of glioma on neurophysiological activity could positively influence higher nervous activity parameters, even in the presence of ongoing tumor growth. This bears significant relevance for malignant gliomas, the most prevalent and lethal type of central nervous system tumors.

Currently, glioma treatment is limited by two main factors: timely detection at onset or relapse and restriction of drugs by the blood-brain barrier from entering the brain and influencing tumor growth. Our study opens up prospects for studying the effectiveness of utilizing low doses of antitumor drugs of various natures with targeted delivery using neurocompatible scaffolds. Despite the fact that the approaches applied in this study turned out to be suboptimal, we were able to show the possibility of preserving mnestic functions and neurological status when modeling highly malignant glioma. Also of extreme interest are data from monitoring the content of UCNPs in the postoperative window. Using non-invasive monitoring methods, it was possible to show that nanoparticles measuring 75 ± 15 nm completely leave the hyaluronic scaffold in less than 14 days. At the same time, the use of scaffolds based on methacrylated hyaluronic acid impregnated with UCNPs allows one to avoid the formation of post-traumatic edema and necrotic changes. Thus, the use of UCNPs in rate calculations makes it possible to determine the rate of release of substances of different molecular weights from the scaffold.

## 5 Conclusion

Scaffolds based on methacrylated hyaluronic acid have demonstrated their efficacy as a drug and UCNPs delivery system for gliomas. Utilizing the aggressive GL261 glioma cell line as a model, it has been established that these hyaluronic scaffolds possess the capacity to ameliorate neurological disorders. An intriguing aspect to consider is that, owing to their photopolymerization-based production, the inclusion of UCNPs within the scaffold composition significantly influences its biological properties, leading to the formation of primary disruption centers during the polymerization process. Consequently, following transplantation, these constructs disintegrate into distinct segments, augmenting the release of UCNPs into the surrounding tissue and concurrently reducing postoperative damage to brain tissue. Moreover, the study has demonstrated the potential for restraining glioma development in the experimental GL261 model through the use of temozolomide. The invasive tumor growth in the SC + TMZ and SC + TMZ + UCNPs groups was less intense compared to the other experimental groups. These findings unveil promising prospects for the application of photopolymerizable biocompatible scaffolds in the realm of neuro-oncology.

## Data Availability

The raw data supporting the conclusions of this article will be made available by the authors, without undue reservation.
