# Supplementary material for: Enhancing glioma treatment with 3D scaffolds laden with upconversion nanoparticles and temozolomide in orthotopic mouse model
Source: Front Chem. 2024 Oct 21;12:1445664. doi: 10.3389/fchem.2024.1445664 (PMC11532134; doi:10.3389/fchem.2024.1445664)
Supplement: Supplementary file 1 [file DataSheet1.docx]

### Supplementary Material

### Whole-body imaging

Animal scanning was implemented with use of a Miniscan scanning head with mirrors powered by galvanic drivers (Raylase, Germany), so the beam of 980 nm semiconductor laser ATS-S4000-200-AMF-980-5-F200 (Semiconductor Devices, Russia) was capable to scan the whole area of interest, which was set by software. UCNP photoluminescence detection was carried out by the Falcon digital EMCCD camera (Raptor, Ireland) carrying an objective with F/0.95-16 iris range (GOYO, Japan). Also the system was equipped with Semrock interference filters installed before the objective. These filters possess the selective transmittance *i.e.* more than 95% of radiation in 485-831 nm range is transmitted, while radiation with a wavelength greater than 980 nm is cut off. These filters allowed cutting off the scattered or reflected radiation of the excitation laser.


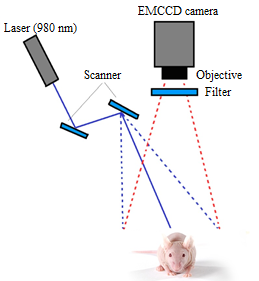


**Figure S1.** Schematic diagram of an optical whole-animal imaging system for UCNPs visualization

Epiluminescent images were obtained while animals were fixed on trays with use of an adhesive tape. Photoluminescent images of the animals were obtained in the range of 485–831 nm under excitation at 980 nm. The excitation light power was 0.77 W, the exposure time was 60 s. Image analysis was performed using ImageJ 1.47v software (National Institute of Health, USA).
